# Exploring the Unknown: Appreciating the Challenges of Non-compaction Cardiomyopathy

**DOI:** 10.7759/cureus.61142

**Published:** 2024-05-26

**Authors:** Muhammad Ibraiz Bilal, Fawwad A Ansari, Muhammad Umer Riaz Gondal, Mubashira Aftab, Abdul Moiz Qureshi, Hayah Kassis-George

**Affiliations:** 1 Internal Medicine, Allegheny Health Network, Pittsburgh, USA; 2 Internal Medicine, Piedmont Athens Regional Medical Center, Athens, USA; 3 Internal Medicine, Tower Health Reading Hospital, West Reading, USA; 4 Internal Medicine, Fazaia Medical College, Islamabad, PAK; 5 Internal Medicine, University of South Alabama, Mobile, USA; 6 Cardiology, Allegheny Health Network, Pittsburgh, USA

**Keywords:** echocardiogram, cardiac mri, anticoagulation, arrythmia, left ventricular non-compaction cardiomyopathy

## Abstract

Left ventricular non-compaction cardiomyopathy (LVNC), or non-compaction cardiomyopathy (NCCM), is defined by pronounced left ventricular trabeculations and deep intertrabecular recesses connecting with the ventricular cavity. Patients with NCCM can be asymptomatic or have severe complications, including heart failure, arrhythmias, thromboembolism, and sudden cardiac death. Our case discusses a patient with shortness of breath who was found to have a newly decreased ejection fraction. The workup revealed non-ischemic cardiomyopathy and cardiac MRI showed hyper-trabeculations consistent with NCCM. The patient was started on oral anticoagulation and guideline-directed medical therapy (GDMT) and discharged with an event monitor. NCCM stands as a relatively rare and enigmatic condition, often veiled in ambiguity. The absence of standardized diagnostic and management protocols further complicates its clinical landscape. While echocardiography is the primary diagnostic tool, its tendency for under-diagnosis poses a significant challenge. Conversely, advanced imaging modalities like cardiac MRI may lead to instances of overdiagnosis. Treatment approaches are non-specific, incorporating GDMT, anticoagulation, implantable cardioverter-defibrillator placement, and genetic testing paired with counseling. Prioritizing genetic research is crucial to uncover tailored therapeutic interventions. Establishing consensus guidelines and refining diagnostic accuracy are pivotal steps toward mitigating the risks associated with under and over-diagnosis.

## Introduction

Non-compaction cardiomyopathy (NCCM) was documented in the literature approximately 80 years ago [1]. In 2006, the American Heart Association formally recognized NCCM as a distinct subtype of cardiomyopathy [2,3]. Despite its acknowledgment, it continues to be poorly understood [4]. Estimates suggest that left ventricular non-compaction cardiomyopathy (LVNC) affects approximately 0.01% to 0.27% of the general population and, in most cases, is hypothesized to result from incomplete left ventricular compaction during embryogenesis [1,2]. In the embryo, muscle tissue adopts a sponge-like structure, allowing cells to receive oxygen and nutrients through blood flow in endothelial spaces due to the absence of coronary and sinusoidal circulation. Typically, myocardial compaction occurs early progressing from basal to apical segments and septal to lateral walls. In congenital NCCM, genetic or epigenetic factors are believed to disrupt this compaction process, resulting in a myocardium with two layers: a compact epicardium and a honeycomb-like endocardium with extensive trabeculation and deep intertrabecular recesses [3]. In adults, LVNC can be isolated, non-isolated (with additional cardiac or syndromic diseases), or acquired (developing in previously unaffected individuals and potentially reversible). The prognosis for adult LVNC is uncertain, as many experience a benign course [5]. It is the abnormal structure of the myocardium that leads to the various manifestations of this disease including heart failure, arrhythmias, thromboembolism, and sudden cardiac death [1]. We present a case of symptomatic NCCM in an older adult that presents as heart failure. This case emphasizes the need for standardized assessment and treatment of this less prevalent medical condition in adults.

## Case presentation

A 70-year-old male with a past medical history significant for osteoarthritis and pre-diabetes presented to the hospital via emergency medical service (EMS) for worsening shortness of breath in the last two weeks. He also reports worsening leg swelling for the past few months, with non-compliance to prescribed furosemide. There was no reported orthopnea, paroxysmal nocturnal dyspnea, palpitations, wheezing, cyanosis, or syncopal episodes. Before presentation to the hospital, the patient was prescribed antibiotics by his primary care physician, which did not improve his symptoms. Due to progressive worsening, EMS was called, and the patient was brought to the hospital. 

On presentation, blood pressure was 130/70, heart rate was 77/min, respiratory rate was 33/min, and oxygen saturation was 82% on room air. The patient was in significant respiratory distress with notable involvement of accessory muscles of respiration. Examination revealed bilateral crackles on auscultation along with bilateral lower extremity edema. Labs showed a white blood cell count (WBC) of 12,900/μL, hemoglobin 16.6 g/dl, BUN 27 mg/dl, creatinine 1.3 mg/dl, AST 131 U/L, ALT 110 U/L, Alkaline phosphatase 140 U/L, and brain natriuretic peptide 5020 pg/mL. Venous blood gas showed pH 7.17, pCO_2_ 55.9mmHg, pO_2_ 137mmHg, and HCO_3_ 19.8mmol/L (see Table 1). The EKG showed normal sinus rhythm without ST-segment or T-wave changes. Chest X-ray was consistent with pulmonary edema with small, bilateral pleural effusions.

**Table 1:** Lab results on admission ALT: Alanine transaminase; AST: aspartate transaminase; ALP: alkaline phosphatase; BUN: blood urea nitrogen; BNP: B-type natriuretic peptide

Variable	Labs (on admission)	Reference range
White blood cells (cells/L)	12900	4500 to 11,000
Hemoglobin (g/dL)	16.6	12.0 to 16.0
Creatinine (mg/dl)	1.3	0.5 to 1.1
ALT (IU/L)	110	10.0 to 40.0
AST (IU/L)	131	10.0 to 40.0
ALP (IU/L)	140	30.0 to 120.0
BUN (mg/dl)	27	7.0-20.0
BNP (pg/mL)	5020	100-300
pH	7.17	7.32-7.43
pCO_2_ (mmHg)	55.9	40-50

**Figure 1:**
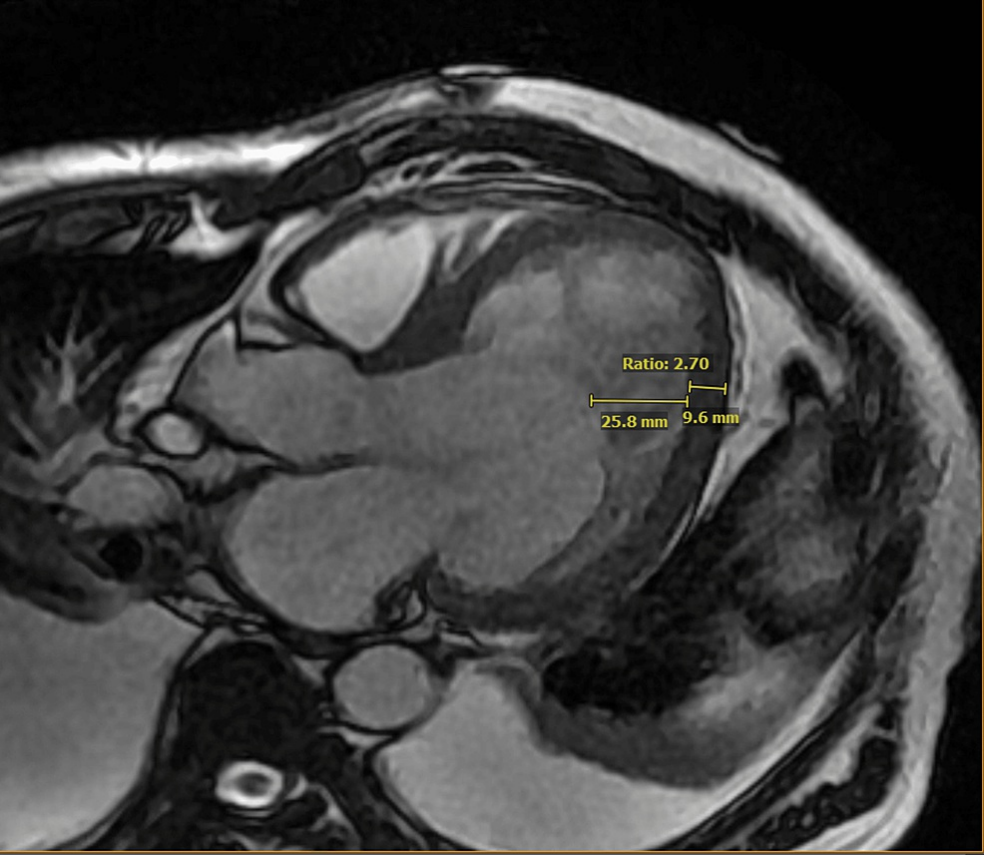
Cardiac MRI showing non-compaction cardiomyopathy This is a long-axis view of cardiac MRI in the end-diastole phase. It shows a non-compaction to compaction ratio of greater than 2.3 thus diagnosing NCCM (Peterson's criteria) NCCM: Non-compaction cardiomyopathy

The patient was admitted to the Cardiac Intensive Care Unit (CICU) and managed aggressively with intravenous furosemide to achieve euvolemia. A transthoracic echocardiogram was obtained, which showed a severely reduced ejection fraction of 10-15%, with grade 2 diastolic dysfunction and mild mitral regurgitation. After achieving volume optimization and transitioning the patient to oral furosemide, GDMT was initiated. A left heart catheterization was performed, which showed minimal non-obstructive coronary artery disease. A cardiac MRI was obtained, and it showed hyper-trabeculation in the inferolateral and anterolateral apical wall segments, with a non-compacted to compacted ratio of more than 2.3 near the mid-lateral wall segment (see Figure 1). The patient was diagnosed with NCCM, according to Petersons' criteria (see Table 2). Oral anticoagulation therapy with a direct oral anticoagulant was initiated due to the elevated risk of thromboembolic events associated with NCCM.

**Table 2:** Different diagnostic criteria for non-compaction cardiomyopathy using echocardiogram and cardiac MRI Our patient was diagnosed using Peterson's criteria for cardiac MRI. Source: [6-9]

Modality	Author (Year)	Diagnostic Criteria
Echocardiography	Jenni et al. (2001)	End-systolic ratio of non-compacted to compacted layer >2:1
Echocardiography	Stollberger et al. (2002)	>3 trabeculations, apical to mid-ventricular, perfused by intraventricular blood
Echocardiography	Petersen et al. (2005)	End-diastolic ratio of non-compacted to compacted layer >2:1
Cardiac MRI	Petersen et al. (2005)	End-diastolic ratio of non-compacted to compacted layer >2.3:1
Cardiac MRI	Jacquier et al. (2010)	Non-compacted mass >20% of total LV mass

Additionally, the patient was discharged with an event monitor and scheduled for close follow-up to evaluate the potential need for cardioverter-defibrillator implantation. The patient remained stable on GDMT outpatient. Given his Holter Monitor did not show significant ventricular arrhythmia, an implantable cardioverter-defibrillator (ICD) was not placed, but the patient did remain on anticoagulation for thromboembolic prophylaxis.

## Discussion

NCCM, also known as spongy myocardium or persistent embryonic myocardium, represents a poorly understood and heterogeneous form of cardiomyopathy [1]. This condition arises from myocardial developmental variations associated with several different mutations. It involves the failure of left ventricle compaction during embryogenesis, leading to ventricular wall thickening and the formation of deep trabecular recesses [1,2].

Patients with NCCM can be asymptomatic or have severe complications, including heart failure, arrhythmias, thromboembolism, and sudden cardiac death [1,5]. Diagnosing non-compaction cardiomyopathy can be challenging due to the absence of clear guidelines, diagnostic criteria, and the heterogeneity of histopathology and presenting symptoms [3]. Moreover, the diagnostic process is further complicated by limited access to tissue samples [5]. 

A transthoracic echocardiogram can be utilized for diagnosing NCCM; however, it is associated with a high rate of false negatives due to limited apex visualization and relatively poor quality [5]. Advanced cardiac imaging such as cardiac MRI is being used increasingly but poses the problem of higher false favorable rates [5]. Other diagnostic tools include left ventricular angiography, electrocardiography, and endomyocardial biopsy, which may help rule out some possible differential diagnoses, including hypertrophic cardiomyopathy, eosinophilic heart disease, LV thrombus, false tendons, aberrant chords, cardiac fibromas, endomyocardial fibrosis, and cardiac metastasis [10].

The foundational approach to treatment aligns with that of other cardiomyopathies, as there is currently no therapy specifically tailored for NCCM. Management primarily involves diuresis to alleviate symptoms of heart failure and guideline-directed medical therapy for the improvement of cardiac function. In select cases, cardiac transplantation may also be considered a viable treatment option [11].

In patients at high risk of arrhythmia, anti-arrhythmic medications are commonly recommended. Additionally, the consideration of ablation therapy for supraventricular and ventricular tachycardia is warranted [10]. ICD placement for patients with reduced ejection fraction can be done based on general guidelines for ICD placement; however, those studies did not involve LVNC cardiomyopathy patients. In this subset of cardiomyopathy, patients with a normal EF are also considered at high risk for arrhythmia in the setting of a prior history of syncope, non-sustained ventricular tachycardia, or a family history of sudden cardiac death. However, definitive guidelines are lacking, and clinicians should weigh the risks and benefits of ICD therapy before proceeding with the intervention [12].

Anticoagulation therapy is recommended since blood pools in the trabecular recesses increase the risk for thromboembolic disease. Generally, anticoagulation with Vitamin-K antagonist is recommended in cases with NCCM and reduced ejection fraction with LVEF < 40% [10]. Patients with atrial fibrillation can also receive newer oral anticoagulation therapy instead of vitamin-K antagonists [10]. If the patient has a normal EF without atrial fibrillation or visible clot, then the benefit of anticoagulation has not yet been demonstrated [12]. Long-term oral anticoagulation is mainly used for patients with atrial fibrillation, impaired left ventricular function, or demonstrated intracardiac thrombi [13]. 

Given the genetic nature of NCCM and its potential familial occurrence, screening family members using electrocardiograms and echocardiograms is advised. Genetic screening is recommended initially in index patients and subsequently in family members to confirm and exclude the presence of NCCM [10].

## Conclusions

NCCM remains a significantly underexplored domain within heart failure medicine. Both the diagnostic and management processes present challenges. The absence of a universally accepted gold-standard diagnostic tool exposes patients to considerable risks, including the potential for under or over-treatment, unnecessary genetic testing, and heightened psychological distress. From the perspective of healthcare providers, it is imperative to conduct pertinent studies examining the risk of arrhythmias and the efficacy of ICDs in managing NCCM. Additionally, there needs to be more data concerning the optimal use of anticoagulation in this patient population, emphasizing the necessity for further research to establish definitive guidelines.
